# Identification of Novel Interaction Partners of Ets-1: Focus on DNA Repair

**DOI:** 10.3390/genes10030206

**Published:** 2019-03-08

**Authors:** Guillaume Brysbaert, Jérôme de Ruyck, Marc Aumercier, Marc F. Lensink

**Affiliations:** University of Lille, CNRS UMR8576 UGSF, Institute for Structural and Functional Glycobiology, F-59000 Lille, France; jerome.de-ruyck@univ-lille.fr (J.d.R.); marc.aumercier@univ-lille.fr (M.A.)

**Keywords:** Ets-1, oncoprotein, DNA repair, biological networks, protein-protein interaction, Residue Interaction Networks

## Abstract

The transcription factor Ets-1 (ETS proto-oncogene 1) shows low expression levels except in specific biological processes like haematopoiesis or angiogenesis. Elevated levels of expression are observed in tumor progression, resulting in Ets-1 being named an oncoprotein. It has recently been shown that Ets-1 interacts with two DNA repair enzymes, PARP-1 (poly(ADP-ribose) polymerase 1) and DNA-PK (DNA-dependent protein kinase), through two different domains and that these interactions play a role in cancer. Considering that Ets-1 can bind to distinctly different domains of two DNA repair enzymes, we hypothesized that the interaction can be transposed onto homologs of the respective domains. We have searched for sequence and structure homologs of the interacting ETS(Ets-1), BRCT(PARP-1) and SAP(DNA-PK) domains, and have identified several candidate binding pairs that are currently not annotated as such. Many of the Ets-1 partners are associated to DNA repair mechanisms. We have applied protein-protein docking to establish putative interaction poses and investigated these using centrality analyses at the protein residue level. Most of the identified poses are virtually similar to our recently established interaction model for Ets-1/PARP-1 and Ets-1/DNA-PK. Our work illustrates the potentially high number of interactors of Ets-1, in particular involved in DNA repair mechanisms, which shows the oncoprotein as a potential important regulator of the mechanism.

## 1. Introduction

Ets-1 (ETS proto-oncogene 1) is a transcription factor involved in specific biological processes related to development, hematopoiesis, angiogenesis or osteogenesis. The gene is usually expressed in delimited time frames associated to these physiological processes but was also found as overexpressed in diseases like rheumatoid arthritis, atherosclerosis and cancers. This association to cancers makes *Ets-1* an oncogene. It is involved more precisely in cancer progression and shows abnormally high levels of expression in invasive cells. Recently, we showed experimentally that Ets-1 interacts with two DNA repair enzymes, namely PARP-1 (poly(ADP-ribose) polymerase 1) and DNA-PK (DNA-dependent protein kinase) and characterized their domains of interaction [1,2]. The ETS (Erythroblast Transformation Specific) domain of Ets-1 can interact with the BRCT (BRCA1 C-terminal) domain of PARP-1 or the SAP (SAF-A/B, Acinus and PIAS) domain of Ku70 (70 kDa unit), a subunit of the DNA-PK DNA repair complex. Moreover, we characterized the binding modes of these interactions considering the domains of the *Homo sapiens* Ets-1, PARP-1 and Ku70 proteins [3]. We found that the binding should occur on the α-helix H1 of the ETS domain, leaving helix H3 available for DNA-binding, and identified a hydrophobic patch in H1 as a central patch of the interaction, which includes three tryptophans (Trp338, Trp356 and Trp361) of Ets-1. Additional residues, Leu342 and Gln339, were also identified in the core of the interface.

DNA repair enzymes act as guardians of the integrity of the genome. Disruption in their pathways have often been associated to disease-related phenotypes, in particular cancers [4,5]. They also play a crucial role in treatments of cancerous cells based on DNA breaks generation like radiotherapy or chemotherapy. Different mechanisms permit to fix damaged DNA depending on the type of lesion. These are divided into two categories, namely single-strand DNA (ssDNA) and double-strand DNA (dsDNA) repair mechanisms. ssDNA itself gathers three types of mechanisms that are base excision repair, nucleotide excision repair and mismatch repair pathways [6]. For dsDNA repair, two general mechanisms, called non-homologous end joining pathways (classical-NHEJ and alternative-NHEJ) and homologous recombination pathways (HR and Single Strand Annealing), are involved, of which the activation depends on the amount of 5′ end resection at the double-strand break [6]. These pathways require many enzymes to work together or sequentially. PARP-1 DNA repair focuses mainly on ssDNA but also promotes the HR pathway and limited end resection for the alternative NHEJ pathway while DNA-PK is known to be involved in dsDNA repair through the classical NHEJ pathway. The catalytic inhibition of PARP-1 in invasive cancerous cells was shown to enhance the accumulation of Ets-1 in the cell and lead to partial cell death [1]. This effect shows an essential role of the partnership between PARP-1 and Ets-1 in invasive cancerous cells. It also raises the question if other DNA repair enzymes could act with Ets-1 in Ets-1 related cancers, which would show this protein as a general perturbator of DNA repair mechanisms.

Considering that Ets-1 is able to bind two different DNA repair enzymes through its ETS domain, we hypothesized that the oncoprotein is able to bind other proteins, potentially involved in DNA repair, as well. We searched for homologs of the ETS, BRCT and SAP domains and identified several candidate binding pairs, of which we then focused on proteins annotated as being associated to DNA repair. We present the method we used based on both sequence and structure information to identify homologs and the different network approaches to identify these new potential partners that are not present in the ego protein-protein network of Ets-1. We discovered new potential interactors of Ets-1 and discussed those associated to DNA repair mechanisms.

## 2. Materials and Methods

**Figure 1:**
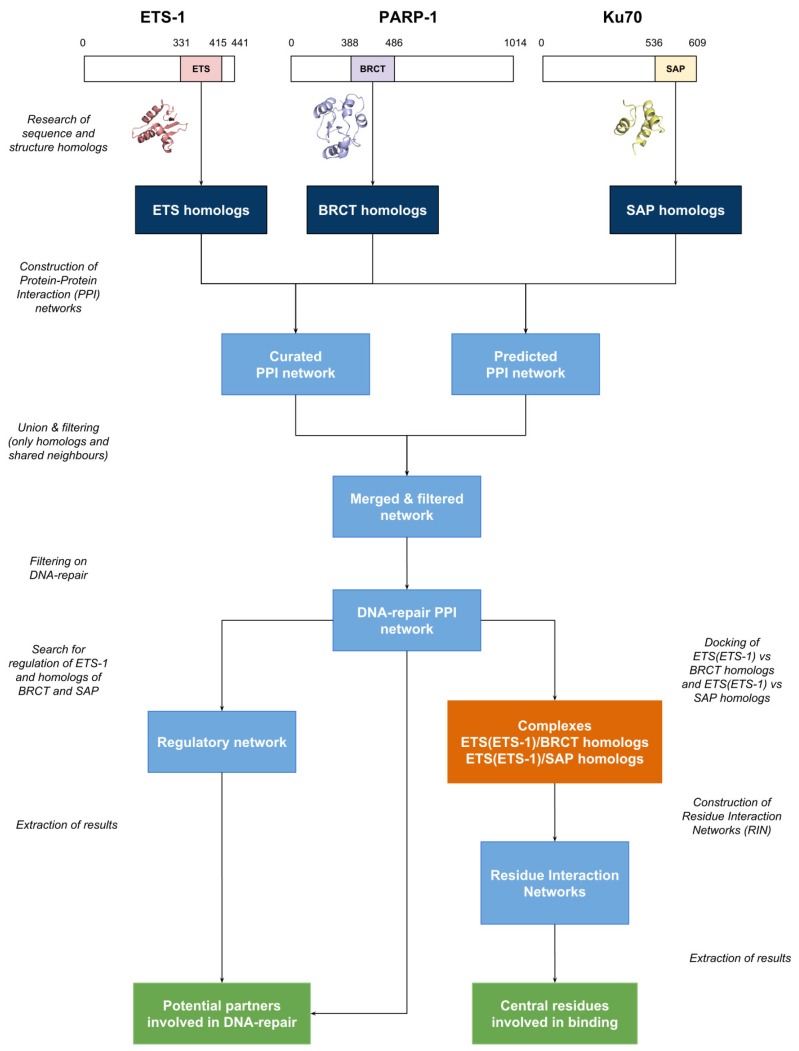
Workflow diagram of the identification of potential partners of Ets-1 involved in DNA repair mechanisms. Homologs are depicted in dark blue, networks in light blue, docking in orange and results in green. Ets-1: ETS proto-oncogene 1; PARP-1: poly (ADP-ribose) polymerase 1; Ku70: 70 kDa unit (or XRCC6: X-ray repair cross complementing 6); ETS: Erythroblast Transformation Specific; BRCT: BRCA1 C-terminal; SAP: SAF-A/B, Acinus and PIAS; PPI: Protein-Protein Interaction.

The workflow of the work described in this article is depicted as a diagram in Figure 1. It is divided into several parts: the research of homologs, the construction of the protein-protein interaction networks (PPIN) and regulatory network (RN), the docking of the domains and the generation of the corresponding Residue Interaction Networks (RIN). Each step is described below.

The list of the potential partners identified with their eventual annotation as belonging to DNA repair pathways is given in supplementary data (Supplementary Table S1).

### 2.1. Homologs

The sequences of the ETS, BRCT and SAP domains of the *Homo sapiens* Ets-1, PARP-1 and Ku70 (also called XRCC6: X-ray repair cross complementing 6) proteins were retrieved from Uniprot:

- ETS: P14921, residues 331 to 415
- BRCT: P09874, residues 388 to 486
- SAP: P12956, residues 536 to 609

The structures of these domains were retrieved from PDB (Protein Data Bank) or modeled and cut with respect to these positions:

- ETS: 1GVJ (331–415)
- BRCT: I-Tasser [7] model (388–486) based on the 2COK structure (BRCT domain which bears a mutation P480S)
- SAP: I-Tasser model (536–609) based on the 1JEQ structure (limited SAP domain)

Sequence homologs were retrieved with BLASTP [8,9] queried with the sequences of the domains on the RefSeq [10] databanks. Only identifiers starting with “NP_” and results with an e-value ≤ 1 × 10 ^−4^ were retained.

Structural homologs were retrieved with BLASTP queried on the PDB databank (e-value ≤ 1 × 10 ^−4^), with Dali [11] and with Kpax 3.1.0 [12] performed on CATH 4.0 [13], SCOP 2.04 [14] and on Dali results (for a filtering of these results). For Dali, only structures with a *Z*-score ≥ 2 were kept. For Kpax, the 200 best structures were conserved and only those with H-score > 0.4. A second order polynomial regression performed on CATH 4.0 and SCOP 2.04 structures shows that such a H-score corresponds roughly to a TM-score > 0.5, which is indicative for fold conservation between output and input [15]. Using the Kpax results, the sequences of the identified homologous structures were submitted to BLASTP to identify sequence homologs of these structures. Only results with an e-value ≤ 1 × 10 ^−4^ and a percentage of identity ≥ 95% were conserved.

Interpro predictions were also retrieved for the respective domains [16] considering the identifiers IPR000418 (ETS), IPR001357 (BRCT) and IPR003034 (SAP). Results were integrated in a common table file per domain and are provided in Supplementary data (Supplementary Tables S2–S4).

For each domain, the sequences of all the homologs were clustered with USEARCH 8/UCLUST [17] with a percentage of identity ≥ 95%. Then, if available in the Protein Data Bank, a *Homo sapiens* structure was retrieved for each cluster. If several were available, the one that covered the entire sequence of the domain, then the one with the best validation criteria was selected as representative crystallographic structure. NMR (Nuclear Magnetic Resonance) structures were considered when no crystallographic ones were available.

### 2.2. Domain-Domain Docking

The protein-protein docking runs between all the BRCT homologs and the ETS domain of Ets-1 on the one side, and between all the SAP homologs and the ETS domain of Ets-1 on the other side were performed with ClusPro with default parameters [18]. Only domains for which the structures were available in the PDB were used in the docking runs. The first model of the first cluster of the resulting binding modes in balanced mode was used for the generation of the Residue Interaction Networks (RIN).

### 2.3. Biological Networks

The Protein-Protein Interactions (PPI) network of Ets-1, PARP-1, Ku70 and the *Homo sapiens* proteins, which bear at least one homologous domain to ETS, BRCT or SAP, was created from the Intact database of curated interactions [19] in the Cytoscape network visualization and analysis software [20,21] using their Uniprot ID. The list of fetched interactions was not filtered. Only the proteins for which interactions were found in Intact appear in the network. Predicted interactions of Ets-1, PARP-1 and Ku70 were retrieved from the FpClass database [22] through the web interface (http://dcv.uhnres.utoronto.ca/FPCLASS/). The metatargetomes of Ets-1, PARP-1, BRCA1, PAXIP1, XRCC1 and MAFG were obtained with the iRegulon app [23] for Cytoscape considering all the predicted regulations (threshold of occurrence count in databases to 1), merged and filtered with Cytoscape core tools. The predicted regulations were assigned based on the gene signatures of GeneSigDB and MSigDB, and on gene sets built with the Ganesh clustering algorithm using default settings to 91 microarray datasets [23]. Gene Ontology Enrichment was performed with the ClueGO app [24] for Cytoscape with default settings, considering biological processes to identify genes/proteins associated to DNA repair (GO:0006281).

Residue Interaction Networks (RINs) were generated for every best model of all the docking runs performed between the ETS domain of Ets-1 and each homologous BRCT or SAP structure (one representative structure of each group). They were created with an in-house C program considering a residue-residue contact when the distance between any atom pair of both residues was found between 2.5 Å and 5 Å. Residue Centrality Analyses (RCA) were performed with the RINspector app [25,26] for Cytoscape. We considered as central a residue with a *Z*-score ≥ 2. More details are given in Appendix A.

### 2.4. Expression Data

Expression data of the MDA-MB-231 cells and the MCF-7 were retrieved from the NCBI (National Center for Biotechnology Information) Gene Expression Omnibus repository [27], from GSE32474 (NCI-60 dataset). They were compared in a differential analysis that was performed between the three replicates of each condition with R/Bioconductor using the affy [28] and limma packages [29]. Data were normalized with RMA (Robust Multi-array Average) and a *t*-test was performed. *p*-values were FDR (False Discovery Rate) corrected for multiple testing. Probes were filtered considering only those with an adjusted *p*-value ≤ 0.01. These data were mapped onto the regulatory network.

## 3. Results

Because Ets-1 can interact with two different domains of two different DNA repair proteins, namely the BRCT domain of PARP-1 and the SAP domain of Ku70 (also called XRCC6), through its ETS domain, we hypothesized that ETS(Ets-1) could also interact with homologous BRCT or SAP domains. Following the workflow depicted in Figure 1, we first identified proteins that contain at least one domain homologous to ETS(Ets-1), BRCT(PARP-1) or SAP(Ku70). In order to then identify which of these are good candidates, we first retrieved experimentally verified interactions between them and then extended the set to predicted interactions of Ets-1, PARP-1 or Ku70. We focused on proteins involved in DNA repair and also considered predicted regulations in order to evaluate a potential interoperability between Ets-1 and a partner. In this regulatory network, we integrated expression data, comparing cancerous cells with high *Ets-1* expression levels to cancerous cells with low *Ets-1* expression levels, and thus, evaluate the effect of Ets-1 on the expression of potential partners. Finally, we performed protein-protein docking between ETS(Ets-1) and identified BRCT and SAP homologs. These were followed by Residue Interaction Networks analyses to compare the predicted binding modes to the established ones for ETS(Ets-1)/BRCT(PARP-1) and ETS(Ets-1)/SAP(Ku70) [3].

### 3.1. Homologs

Knowing that the ETS domain of Ets-1 can bind the BRCT domain of PARP-1 or the SAP domain of Ku70, we searched for sequence and structure homologs of the individual domains of BRCT(PARP-1) and SAP(Ku70), in order to identify new additional binding partners of ETS(Ets-1). ETS(Ets-1) homologs were also searched to identify some of them that are known to bind to BRCT and/or SAP homologs in the following protein-protein interaction network, which would allow to infer a potential binding by ETS(Ets-1) as well. The homologs found are gathered in three different tables in Supplementary data, one for each domain, and clustered in function of their similarity of protein sequences (see Materials and Methods section for details). We assigned a structure to each cluster if one was available in the Protein Data Bank. The number of homologous genes, PDB structures found and clusters of sequences are listed in Table 1. The differences in numbers between genes and clusters can be explained by the fact that one gene can bear several BRCT domains, with amino acid sequences that are more or less similar.

24 BRCT and 10 SAP clusters have a representative PDB structure that was then used in protein-protein docking essays against the ETS domain of Ets-1 (see below).

### 3.2. Protein-Protein Interaction Network

#### 3.2.1. Experimentally Characterized Interactions

**Figure 2:**
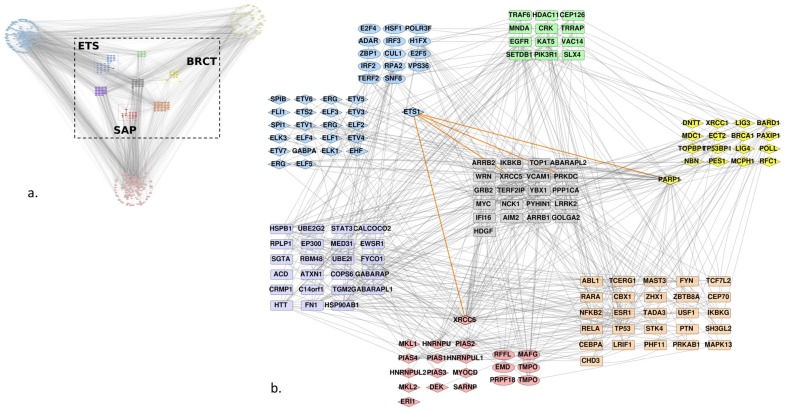
Human protein-protein interaction (PPI) network of Ets-1, PARP-1, Ku70, and proteins that bear a domain homolog to the ETS domain of Ets-1, BRCT domain of PARP-1 and SAP domain of Ku70. (**a**) Global first neighbor PPI network; ETS homologs are colored blue, BRCT homologs are colored yellow and SAP homologs are red; first neighbors of ETS, BRCT and SAP homologs are shown at the extremities of the graph and plotted in another shade of blue, yellow and red, respectively; common interactors between different types of homologs are colored in a mix of colors of the homologs, resulting in green for ETS and BRCT, purple for ETS and SAP, and orange for SAP and BRCT; proteins that interact with at least one protein of each group of homologs are colored grey. (**b**) Central network delimited by the dashed square in (**a**), only interactors that are shared between at least two groups are conserved; diamond forms are the proteins that were annotated by Interpro as containing a ETS, BRCT or SAP domain, ellipses are the additional homologs we have found, using our method; rounded rectangles are identified interactors; orange edges indicate known interactions with Ets-1.

We used the Uniprot identifiers of the Ets-1, PARP-1 and Ku70 (or XRCC6) proteins and of the found homologs to query the Intact database and retrieve the curated interactions between them. Figure 2a shows the resulting network, it presents direct edges between the proteins and their first neighbors. The homologs were gathered in a grid layout, each group being split into two subgroups: those that were known to bear an ETS, BRCT or SAP domain following the Interpro annotation (in diamonds) and the other homologs that we have found with our method. Only the BRCT group did not show any such homologs. Given the complexity of the graph, we decided to focus on the interactors shared by at least two groups of homologs (Figure 2b). Although we did not filter any edges in the graph on the type of interaction, in order to conserve the maximum amount of information on interactors between the different groups, the graph shows very few of them between the individual groups. The majority of interactions retrieved from Intact between the ETS group and the two others were focused on PARP-1 and Ku70 (XRCC6) (highlighted in orange for Ets-1 interactions). Nevertheless, we identified an interaction between the ERG protein of the ETS group and HNRNPU of the SAP group. The other ones were indirect through other proteins.

#### 3.2.2. Predicted Interactions

**Figure 3:**
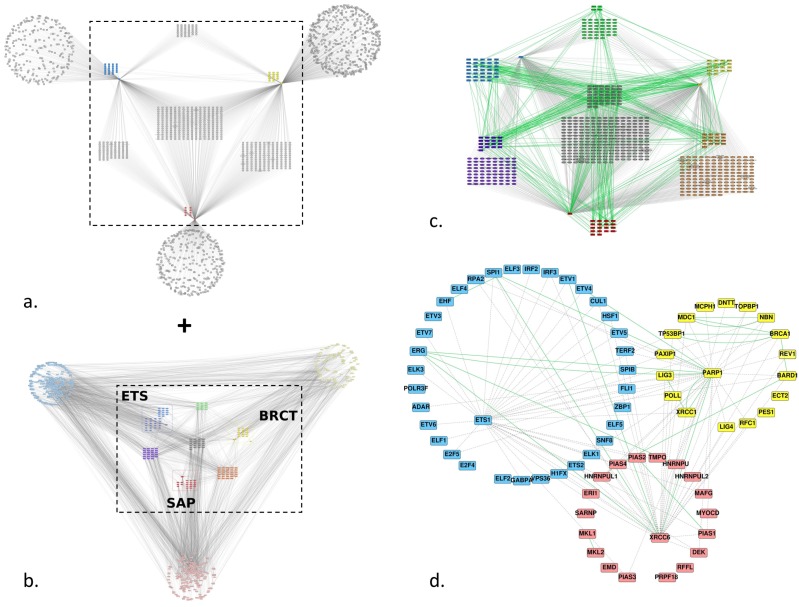
Predicted and experimentally identified protein-protein interaction (PPI) network of Ets-1, PARP-1, Ku70 and proteins that contain one of the ETS, BRCT or SAP homologous domains: (**a**) first-neighbour predicted network of Ets-1, PARP-1 and Ku70 (XRCC6); predictions were retrieved from FpClass; the group of ETS homologs is colored blue, the one of BRCT homologs is colored yellow and the one of SAP homologs is colored red; (**b**) global first neighbor PPI network; this is the same network as in Figure 2a; (**c**) union of the center part of the networks (**a**,**b**), the color code of the nodes is the same as in Figure 2; edges are colored green for experimentally established interactions and dashed/grey for predicted ones; (**d**) the same network as (**c**), keeping only the proteins of the three groups of homologs.

To increase the number of edges between the ETS group and the BRCT and SAP groups, we added predicted interactions from the FpClass database for Ets-1, PARP-1 and Ku70 (XRCC6) (Figure 3a). We conserved only the shared interactors between the three proteins like we did for the three groups of homologs for the Intact PPI network and merged the FpClass and Intact networks (Figure 3c). The final network (Figure 3d) shows only the proteins of the three groups of homologs and depicts additional links between the three groups, in particular between (i) Ets-1 and BARD1, BRCA1, NBN, TP53BP1, XRCC1 of the BRCT homologs, (ii) between RPA2, TERF2, H1FX, E2F5, ELF1, ADAR of the ETS homologs and PARP-1, (iii) between Ets-1 and HNRNPUL1, PIAS4, PIAS2, HNRNPU, MAFG, PIAS1, DEK, PIAS3 of the SAP homologs and (iv) between H1FX, ETS2, TERF2, HSF1, CUL1, IRF3, RPA2 of the ETS homologs and Ku70.

### 3.3. DNA Repair

**Figure 4:**
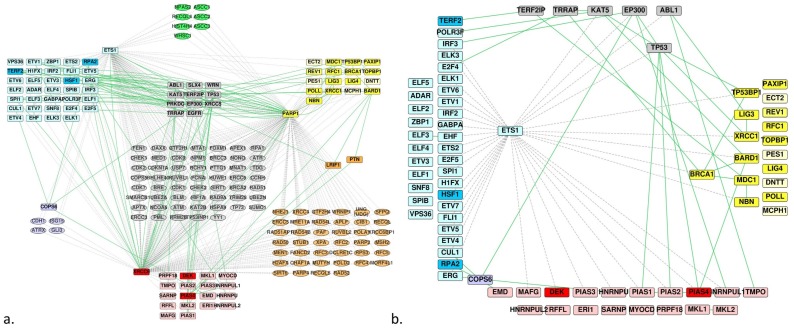
PPI network filtered on proteins involved in DNA repair mechanisms. (**a**) The same network as in Figure 3c but filtered to keep proteins involved in DNA repair and all homologs; the homologs that are not involved in DNA repair are displayed in a lighter shade; edges are colored green for experimentally verified interactions and dashed/grey for predicted ones. (**b**) The same network as in (**a**) but PARP-1 and Ku70(XRCC6) were removed; subsequently, only the proteins that connect a ETS homolog to a BRCT or SAP homolog were conserved, one of the ETS homolog partners being involved in DNA repair.

Considering the predicted and experimentally determined interactions between the homologs of ETS and those of BRCT or SAP, we hypothesized that some of the last two groups could constitute potential partners of the Ets-1 protein, especially those involved in DNA repair mechanisms, seeing that Ets-1 also interacts with PARP-1 and DNA-PK. Focusing on the merged PPI network of the homologs and common partners (Figure 3c), we annotated and filtered the network according to their association to DNA repair activities with the ClueGO app for Cytoscape. We obtained the PPI network depicted in Figure 4a, where we kept all the proteins of the three homologs groups. We filtered the network to conserve (i) the proteins of the three groups of homologs, (ii) the proteins that are not involved in DNA repair but that make bridges between the proteins of the group of the ETS homologs and the proteins of the group of the SAP or BRCT homologs involved in DNA repair and (iii) the proteins of the SAP or BRCT group that are not annotated as belonging to DNA repair mechanisms but that are connected to a protein that is involved in DNA repair and makes a bridge with the ETS group. Because the interactions Ets-1/PARP-1 and Ets-1/Ku70 are already known, we removed them from the network (Figure 4b).

This last network shows 14 candidates for binding to Ets-1 relating to DNA repair mechanism. These include: (i) TP53BP1, XRCC1, BARD1, BRCA1 and NBN for BRCT homologs (predicted by FpClass and involved in DNA repair mechanisms), (ii) DEK and PIAS4 for SAP homologs (same reason), (iii) LIG3, MDC1, EMD, PIAS1, PIAS2, HNRNPUL1 and TMPO (interact with a protein involved in DNA repair that is predicted to interact with Ets-1 or interacts with an ETS homolog).

**Figure 5:**
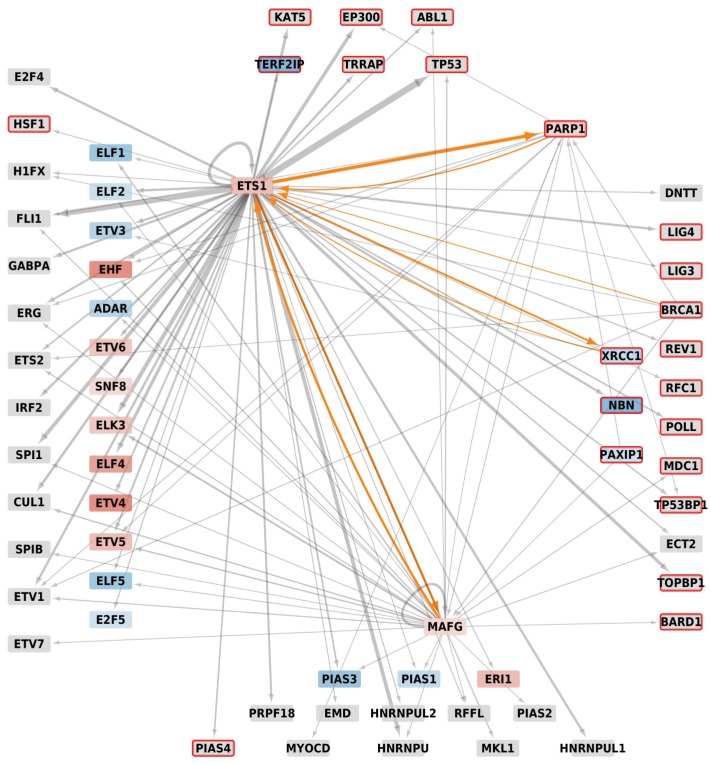
Metatargetome of Ets-1, PARP-1, BRCA1, PAXIP1, XRCC1 and MAFG filtered on the proteins of the network of Figure 4b. Differential expression data between MDA-MB-231 and MCF-7 cells were mapped onto the network; only signals with adjusted *p*-value ≤ 0.01 were kept; nodes are colored as a gradient of the log_2_ fold-change from blue (log_2_(Fold Change) ≤ −2) to white (log_2_(FC) = 0) and red (log_2_(FC) ≥ 2); grey nodes do not show significantly different signals; the width of arrows relates to the number of occurrences in databases found by iRegulon, the larger, the higher number; on the left stand the ETS homologs, at the bottom the SAP homologs, on the right the BRCT homologs and top shows the other proteins of the Figure 4b; proteins that are associated to DNA-repair have a red border; orange arrows are those that involve a regulation by Ets-1, eventually reciprocal.

Several homologs being transcription factors, we built their metatargetomes with the iRegulon app for Cytoscape to know if these identified potential interactors were amenable to regulation by Ets-1, with an eventual reciprocal regulation. We mapped expression data resulting of a differential analysis between MDA-MB-231 cells versus MCF-7 cells and filtered signals to keep only those that showed an adjusted *p*-value ≤ 0.01. The MDA-MB-231 cells were used as a model of invasive cancer cells in which high levels of expression of *Ets-1* are measured, while MCF-7 cells are non-invasive cells that show low levels of expression of *Ets-1*. With iRegulon, we found the metatargetomes of Ets-1 (9904 predicted regulations by Ets-1), BRCA1 (855 predicted regulations), PARP-1 (1529 predicted regulations), PAXIP1 (336 predicted regulations), XRCC1 (302 predicted regulations) and MAFG (4206 predicted regulations). We merged and filtered them conserving only the subset of proteins of Figure 4b and mapped expression data onto the resulting network (Figure 5).

In this regulatory network, only *PARP-1*, *XRCC1*, *NBN* and *PAXIP1* appear to show a significant log_2_(Fold Change) signal and are involved in DNA repair mechanisms. Their differential signal is negative except for *PARP-1*. Therefore, while *PARP-1* was overexpressed, *XRCC1*, *NBN* and *PAXIP1* were repressed in MDA-MB-231 compared to MCF-7 which indicates that the amount of the corresponding proteins should be lowered as well, thus resulting in a limited repair activity, while *Ets-1* gene expression is higher. Nevertheless, the three DNA repair genes still showed a relatively high signal of expression compared to the other genes of the samples (percentile rank > 50% within the samples) and the amount of Ets-1 proteins is elevated in MDA-MB-231. The other homologs do not show differential expression (depicted in grey in Figure 5); however, they all show relatively good levels of expression in MDA-MB-231 (percentile rank > 50% within the samples). Thus, we did not see a strong signal of regulation by Ets-1 of the new potential partners involved in DNA repair. Consequently, any correlated activity between Ets-1 and the identified DNA repair proteins is likely to result from an interaction at the protein level, which cannot be identified with the gene signatures used by iRegulon for the construction of the regulatory network. These interactions were made possible because *Ets-1* and these DNA repair genes showed good levels of expression. Furthermore, two DNA repair proteins appear as exceptions in this regulatory network, namely PARP-1 and XRCC1. These proteins, amenable to regulation by Ets-1, could also in turn regulate Ets-1, highlighting a tight connection between them. MAFG also shows such a reciprocal regulation with Ets-1.

### 3.4. Protein-Protein Docking and Residue Interaction Networks

To confirm the relevance of these potential candidates, we performed docking runs between the ETS domain of Ets-1 and the BRCT or SAP homologous domains of each cluster identified. The representative structures of the BRCT or SAP clusters were used and docking only performed for those clusters for which a structure was available (24 for BRCT homologs, 10 for SAP homologs). Subsequently, we ran Residue Centrality Analyses on the best model of each run to identify central residues at the interface and to compare them to the residues identified in [3] for the binding of ETS(Ets-1)/BRCT(PARP-1) and ETS(Ets-1)/SAP(Ku70) (see Appendix A). We calculated these for four sets of structures: all the structures available for the BRCT homologs and the SAP homologs, and the two subsets of those that are associated to DNA repair mechanisms. Table 2 shows the residues of the ETS(Ets-1) domain that were found as central in at least two structures and that belong to the interface. Three residues are found in all the groups, namely Trp338, Trp356 and Trp361, with a systematic presence of Trp361 at the interface and with a high *Z*-score in all the structures (except one *Z*-score ≥ 2 for one BRCT structure). Leu342 also appears in the ETS/SAP binding pairs, while Gln339 appears in the ETS/BRCT pairs. With the exception of Thr346, these residues are part of a hydrophobic patch that was identified previously [3]. All the first poses of the docked structures of the homologs show an interaction with the α-helix H1 of ETS, involving the same ETS residues as for the interaction with PARP-1 and Ku70. This is a strong argument that the identified homologs can be considered good candidates for the binding to the ETS domain of Ets-1.

## 4. Discussion

We recently demonstrated that the ETS domain of Ets-1 can interact with the BRCT domain of PARP-1 or with the SAP domain of Ku70, a subunit of the DNA-PK complex, both of them being involved in DNA repair mechanisms [1,2]. Based on the similarity of binding modes [3], we searched for sequence and structure homologs of these ETS, BRCT and SAP domains, in order to identify new potential partners of the Ets-1 oncoprotein, focusing on those potential partners that are involved in DNA repair mechanisms. We identified 42 genes that express a protein domain homologous to the ETS domain of Ets-1, 19 genes for the BRCT domain of PARP-1 and 19 genes for the SAP domain. We assembled all the interactions known in the Intact database between these homologs and created a protein-protein interaction network. The network was extended with predicted interactions from the FpClass database. We then focused on proteins involved in DNA repair mechanisms. At this level, we identified 14 relevant candidates for binding to the ETS domain of Ets-1, which are (i) BRCT homologs: TP53BP1, XRCC1, BARD1, BRCA1, NBN, LIG3 and MDC1, and (ii) SAP homologs: DEK, PIAS4, EMD, PIAS1, PIAS2, HNRNPUL1 and TMPO. We finally performed protein-protein docking simulations with the representative PDB structures for each cluster of BRCT and SAP homologs versus the ETS domain of Ets-1. We found the binding modes of the different homologs (to ETS) to be compatible with the ones established in [3], involving the α-helix H1 of Ets-1 and a hydrophobic patch centered around Trp361.

Among these, BRCA1 was not referenced in Intact at the time we performed this work, but found as a predicted interactor by FpClass. However, this interaction has been identified experimentally as a partner of Ets-1 [30]. It was shown that full-length Ets-1 interacts with BRCA1 and that the ETS domain of Elk-1, a member of the ETS family, interacts with BRCA1, which stands as a validation of our approach. BRCA1 has an activity in DNA repair but more specifically in double-stranded DNA-breaks repair by the homologous recombination repair mechanism. Like PARP-1, it contains a BRCT domain as potential binding partner for Ets-1. Legrand et al. [1] showed that when PARP-1 is catalytically inhibited by PJ-34, MDA-MB-231 cells showed about half of the cells undergoing necrosis. Under PARP-1 inhibition, unrepaired single-stranded DNA breaks lead to double-stranded breaks during replication, which can be repaired, amongst others, by enzymes like BRCA1 and BRCA2. Defects in these genes are commonly associated with an increased risk factor for breast cancer [31]. Therefore, BRCA1 interaction with Ets-1, which could happen through one of its BRCT domains, would have an effect on double-stranded repair, thereby explaining the necrosis ratio of the MDA-MB-231 cells under PJ-34 treatment. This idea goes in the same direction as the model of synthetic lethality mechanisms, which shows that PARP inhibitors kill tumors defective in BRCA1 or BRCA2 genes [6,31]. A known partner of BRCA1, namely BARD1 (BRCA1-associated RING domain protein 1), contains two additional BRCT domains that could be targeted by Ets-1, offering multiple binding possibilities of Ets-1 to the BRCA1/BARD1 complex. In BARD1, three point mutations have been found in breast and/or ovarian cancer susceptible patients [32]. One of these, R658C, is located in the first BRCT domain, close to the predicted interaction site with Ets-1.

Likewise, while not found in Intact, but predicted by FpClass, we identified TP53BP1 (TP53 Binding-protein 1). While TP53, a well-known tumor suppressor whose several mutations are associated to various forms of cancer [4], is known to interact with Ets-1 [33,34], nothing is currently known about a possible interaction between Ets-1 and TP53BP1. However, certain mutations in TP53BP1 can also be associated to various cancers, more precisely to breast and skin cancers [4]. Obviously, TP53BP1 binds TP53, but more importantly, this protein is involved in the classical NHEJ pathway, similar to DNA-PK. The binding of Ets-1 to TP53BP1 (through its BRCT domain) could have an impact on the interplay between Ets-1 and TP53. Our data shows the possibility for these three proteins to interact simultaneously: the superpositions of the five best representative docking poses of one TP53BP1 BRCT domain versus the ETS domain of Ets-1 on the crystal structure of the BRCT domains of TP53BP1 bound to TP53 (PDB ID: 1KZY [35]) shows that the ETS domain of Ets-1 could bind the second BRCT domain of TP53BP1 without clashing with TP53 (Supplementary Figure S1).

**Figure 6:**
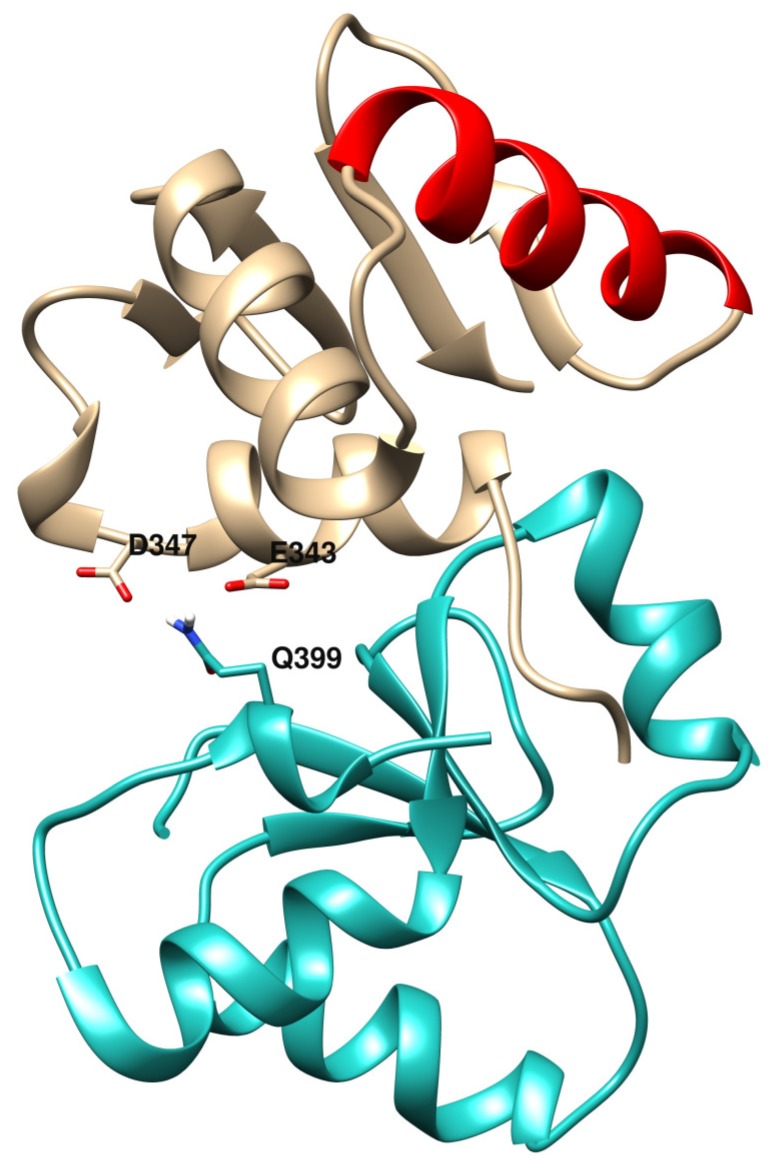
First model of the docking of the ETS domain of Ets-1 (top) and the first BRCT domain of XRCC1 (bottom). At the interface, the Q399 variant of XRCC1 is depicted in stick as well as the two negatively charged amino acids D347 and E343 of ETS. The DNA-binding helix H3 of ETS(Ets-1) is colored red.

Concerning NBN and XRCC1, gene expression levels measured in MDA-MB-231 (in which Ets-1 levels are high) are lower than in MCF-7 (in which Ets-1 levels are low) and Ets-1 is a potential regulator of them. We see in our analysis that when *Ets-1* is highly expressed in the invasive cells, these DNA repair genes are repressed, which should have consequences on the ability of the cells to repair their DNA lesions. However, even if repressed, the two genes still show relatively good levels of expression and probably still a reasonable amount of their proteins in the cells. Therefore, they could interact with Ets-1 through its ETS domain and these interactions could play a role in DNA repair mechanisms. Indeed, it was shown that mutations in the *NBN* gene are associated to an increase of a risk of breast cancer through the double-stranded break repair mechanism [36]. XRCC1 mutants are also associated with breast cancer [5,37]. For instance, mutation R399Q was found to be positively associated to breast cancer, which is located at the end of the first BRCT domain of XRCC1 and at the interface of the best binding mode predicted by Cluspro in the ETS(Ets-1)/BRCT(XRCC1) docking run (Figure 6). The BRCT domain used for the docking (PDB ID: 2D8M) bears this Gln variant at position 399. With an Arg at this position, the affinity between the two domains could be increased, with potential stronger binding for the R399 variant through electrostatic interactions with both E343 and D347 of the ETS(Ets-1) domain. In this model, the R399Q variant destabilizes the interaction between ETS and XRCC1. Moreover, it is known that XRCC1 interacts with LIG3 for ligation to DNA through its second BRCT domain [38,39], which is also involved in homodimeric formation, letting space for a potential binding of Ets-1 to the first BRCT domain. Furthermore, it would be possible that ETS(Ets-1) binds the second BRCT domain of XRCC1 potentially preventing the formation of functional oligomers. In addition to this potential interaction between Ets-1 and XRCC1, Figure 5 presents a reciprocal regulation between them, which shows a tight connection between the two proteins, as is the case for Ets-1 and PARP-1. Ets-1 could even interact with LIG3 through its respective BRCT domain, enabling the oncoprotein at several levels to disrupt the ligase activity of this complex in base excision repair or alternative NHEJ pathways.

The ligase LIG4, which contains two BRCT-homologous domains, is involved in the ligation process of the classical non-homologous end-joining pathway. While the paragraphs above show an established (Intact) or predicted (FpClass) interaction at the protein level, LIG4 does not show any interaction in the PPI network we created (Figure 4b). However, either BRCT domain of LIG4 could potentially be targeted by the ETS domain of Ets-1. It has been shown by Wu et al. [40] that XRCC4 interacts with LIG4 through the second BRCT domain of LIG4 (and the inter-BRCT linker region), leaving the first BRCT domain available for binding to another molecule. However, Ets-1 could also directly bind the second BRCT domain of LIG4, thereby preventing its interaction with XRCC4. Likewise, the DNA polymerase lambda, shortly called POLL, plays a role in several DNA repair pathways, especially in the base excision repair and classical non homologous end joining pathways, for what is currently known [6,41]. No known or predicted interaction with Ets-1 can be found in its PPI network; however, POLL is known to interact with the XRCC4-LIG4 complex through its BRCT domain [42] and this interaction could potentially be disrupted by intervention of Ets-1 through its ETS domain.

Lastly, TOPBP1, involved in DNA repair and DNA replication, contains as many as eight BRCT domains, making it a large target for Ets-1 to bind at least to one of them. Deregulated activity of TOPBP1 has been associated to cancer, in particular breast cancer, and it is considered a potential target for cancer therapy [43,44]. Similarly, PAXIP1 contains many BRCT domains (six domains) that might be targeted by Ets-1.

Initially, we considered only known protein interaction pairs, including the interactions occurring through homologous ETS and homologous BRCT or SAP. In our request to the Intact database, we only found ERG of the ETS family interacting with the HNRNPU protein of the SAP homologs. However, HNRNPU is not known to be involved in DNA repair. On the other hand, we already mentioned that Elk-1, also of the ETS family, is known to interact with BRCA1. The interaction network shows that several of the ETS homologs, including ERG, are known to interact with PARP-1 or DNA-PK [45]. Therefore, we cannot exclude a competitive binding between Ets-1 and members of the ETS family. In cells overexpressing Ets-1, such as MDA-MB-231 cells, interactions with Ets-1 could be favored because of the very high amount of the Ets-1 protein. The BRCT and SAP homologs that we found as potential interactors with Ets-1 are more or less divergent in sequence composition, which inevitably leads to various affinities in binding.

In Ets-1, the ETS domain contains two auto-inhibitory regions composed of four helices (HI-1, HI-2, H4 and H5) that fold onto the ETS domain. Moreover, it possesses a Serine Rich Region (SRR) upstream in the sequence (279-295) with some of them prone to phosphorylation, which may regulate the auto-inhibition [46]. The binding between the H1 helix and the auto-inhibitory domains is weak and can be easily destabilized [47]. Here, we limited our study to the ETS domain alone, without these regulatory domains, corresponding to the situation where the auto-inhibition is already released. In our context, this disruption would be provoked by the identified interaction partners prior to their binding to H1. It has been shown that phosphorylation of the SRR domain enhances the auto-inhibition [46,48], highlighting an important role of post-translational modifications in the binding to the ETS domain. Therefore, taking into account the effect of potential post-translational modifications on binding would be of high interest in a further extensive study.

Considering proteins that might bind to the ETS domain of Ets-1, that are involved in different pathways of DNA repair, and this at different levels, it raises the question if Ets-1 could act as a general regulator or perturbator of DNA repair, in particular in invasive cancerous cells where the gene is overexpressed. Consequently, it could be of high interest to design inhibitors that alter the interaction between the ETS domain of Ets-1 and its partners. This inhibition may have an impact on DNA repair activity in invasive cancerous cells.

## Figures and Tables

**Table 1:** Number of homologous genes, PDB structures and clusters of sequences found for the ETS, BRCT and SAP domains. Each cluster gathers sequences which share at least 95% identity

	Homologs		
Domain	Number of Genes	Number of PDB Structures	Number of Sequence Clusters
ETS	42	23	38
BRCT	19	24	30
SAP	19	10	19

PDB: Protein Data Bank; ETS: Erythroblast Transformation Specific; BRCT: BRCA1 C-terminal; SAP: SAF-A/B, Acinus and PIAS.

**Table 2:** Residues of the ETS domain of Ets-1 found at the interface that have a Residue Centrality Analysis *Z*-score ≥ 2 at least twice in the set of structures. The table shows the number of times the residue is found (written “Nb”) at the interface and the number of times it has a *Z*-score ≥ 2. It presents the results for four sets of structures: all ETS/SAP homologs (10 structures), ETS/SAP homologs involved in DNA repair (5 structures), all ETS/BRCT homologs (24 structures) and ETS/BRCT homologs involved in DNA repair (18 structures).

	ETS/SAP Homologs—All	
Residue	Nb Interface	Nb *Z*-Score ≥ 2
Trp361	10	10
Leu342	10	5
Trp338	10	5
Trp356	10	3
	**ETS/SAP homologs—DNA repair**	
Trp361	5	5
Leu342	5	2
Trp338	5	2
Trp356	5	2
	**ETS/BRCT homologs—All**	
Trp361	24	23
Trp338	24	9
Thr346	24	6
Gln339	24	5
Trp356	24	5
Phe414	24	3
Glu343	24	2
	**ETS/BRCT homologs—DNA repair**	
Trp361	18	17
Trp338	18	7
Thr346	18	6
Gln339	18	4
Trp356	18	3
Phe414	18	3

**Figure A1:**
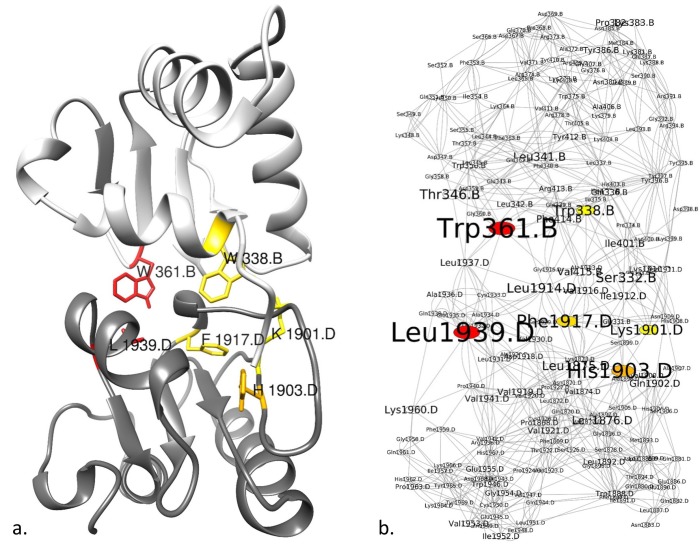
Residue Centrality Analysis (RCA) performed on the Residue Interaction Network (RIN) generated from the best docking pose of the ETS domain of Ets-1 (PDB ID: 1GVJ) and the second BRCT domain of TP53BP1 (PDB ID: 1GZH). (**a**) Structure of the best docking pose calculated by ClusPro; at the top in light grey is ETS(Ets-1); at the bottom in dark grey is BRCT(TP53BP1); central residues at the interface are depicted with atomic side chains in a gradient of color from yellow (*Z*-score = 2) to red (*Z*-score ≥ 4); (**b**) RIN generated from the structure (**a**); central residues are those depicted in (**a**) with the same color code, the bigger the node and label sizes, the higher the *Z*-score, other nodes are white.

## Supplementary Materials

The following are available online at https://www.mdpi.com/2073-4425/10/3/206/s1, Figure S1: Structure of the two BRCT domains of TP53BP1 bound to TP53 and ETS(Ets-1); Table S1: List of all homologs (Uniprot ID, Gene name and annotation of those involved in DNA repair); Table S2: Annotated list of the homologs of the ETS domain of Ets-1; Table S3: Annotated list of the homologs of the BRCT domain of PARP-1, Table S4: Annotated list of the homologs of the SAP domain of Ku70.

## Appendix A

Residue Interaction Networks (RINs) are networks generated from a PDB structure where nodes are residues and any edge is a detected interaction between a pair of residues inside the structure. An interaction was defined as any atom contact pair between 2.5 Å and 5 Å. The RINs were generated for each best docking pose between the ETS domain of Ets-1 and a BRCT or SAP homolog. Consequently, 24 RINs were generated for ETS(Ets-1)/BRCT homologs and 10 RINs for ETS(Ets-1)/SAP homologs. Once created, a Residue Centrality Analysis was performed, which is based on the change in average shortest path length under removal of each node. The shortest path between two nodes in a network is defined as the path with the least amount of edges connecting the first node to the second one, the shortest path length then being the number of edges. The average shortest path length (ASPL) is the average of shortest path lengths of all possible pairs of nodes in a network. In the residue centrality analysis, a per-node *Z*-score is calculated as follows:

(1) $$z_{k}=\frac{∆L_{k}-\bar{∆L}}{\sigma }$$

where $∆L_{k}$ is the change of the ASPL under removal of node k, $\bar{∆L}$ is the change of the ASPL under node removal averaged over all protein residues and σ is the corresponding standard deviation.

We considered those nodes that exhibited a value ≥ 2 as central (for more details, see [25,49]). For comparison to the binding modes identified in [3], we focused on central residues at the interface between the two chains. Figure A1 illustrates an example of a RIN generated from the best docking pose of ETS(Ets-1)/BRCT(TP53BP1).
