# Supplementary material for: Identification of Novel Interaction Partners of Ets-1: Focus on DNA Repair
Source: Genes (Basel). 2019 Mar 8;10(3):206. doi: 10.3390/genes10030206 (PMC6470857; doi:10.3390/genes10030206)
Supplement: Supplementary file 1 [file genes-10-00206-s001.zip › Supplementary_Figures.pdf]

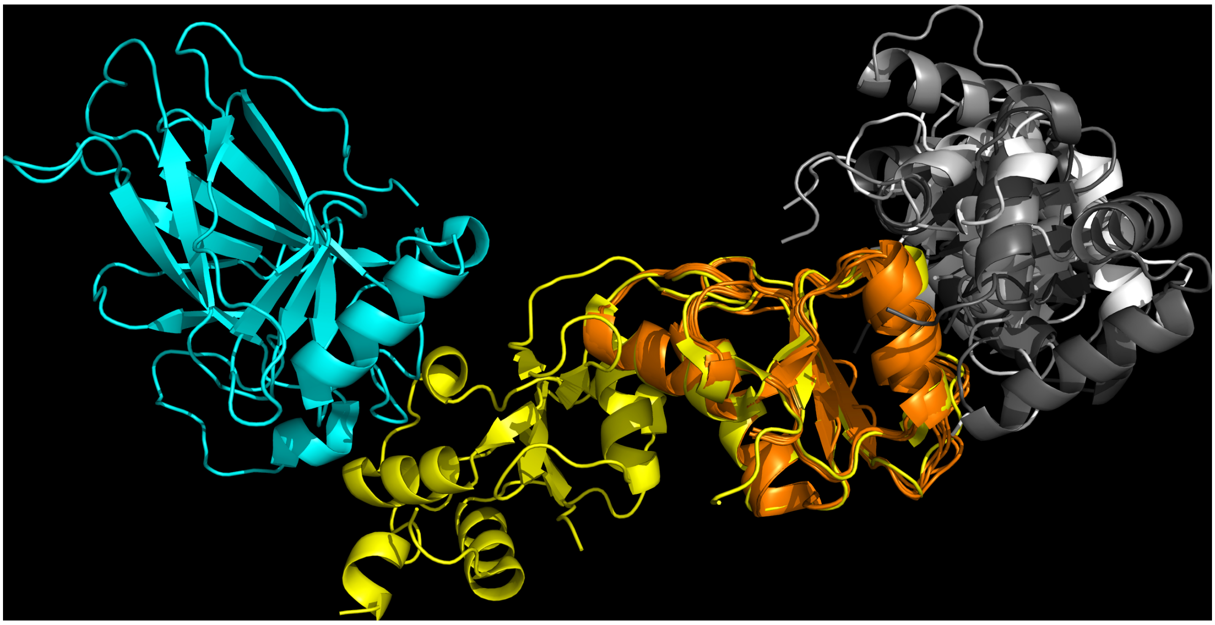

Figure S1. Structure of the two BRCT domains of TP53BP1 (yellow) bound to TP53 (PDB ID: 1KZY) (cyan) with the superposition of the five first docking poses of one BRCT domain of TP53BP1 (orange) vs the ETS domain of Ets-1 (5 shades of grey), in cartoon representation.
